# Structural Consequences of Introducing Bioactive Domains to Designer β-Sheet Peptide Self-Assemblies

**DOI:** 10.1021/acs.biomac.3c00962

**Published:** 2024-02-26

**Authors:** Alicia S. Robang, Abhishek Roy, Joseph B. Dodd-o, Dongjing He, Justin V. Le, Andrew C. McShan, Yuhang Hu, Vivek A. Kumar, Anant K. Paravastu

**Affiliations:** † School of Chemical and Biomolecular Engineering, Georgia Institute of Technology, Atlanta, Georgia 30332, United States; ‡ Department of Biomedical Engineering, New Jersey Institute of Technology, Newark, New Jersey 07102, United States; § George W. Woodruff School of Mechanical Engineering, Georgia Institute of Technology, Atlanta, Georgia 30332, United States; ∥ School of Chemistry and Biochemistry, Georgia Institute of Technology, Atlanta, Georgia 30332, United States; ⊥ Parker H. Petit Institute for Bioengineering and Biosciences, Georgia Institute of Technology, Atlanta, Georgia 30332, United States; # Department of Chemicals and Materials Engineering, New Jersey Institute of Technology, Newark, New Jersey 07102, United States; ∇ Department of Biology, New Jersey Institute of Technology, Newark, New Jersey 07102, United States

## Abstract

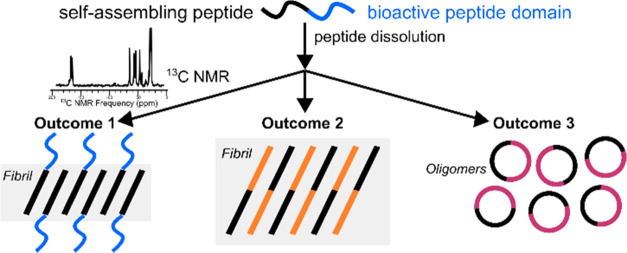

We applied solid- and solution-state nuclear magnetic resonance spectroscopy to examine the structure of multidomain peptides composed of self-assembling β-sheet domains linked to bioactive domains. Bioactive domains can be selected to stimulate specific biological responses (*e.g*., *via* receptor binding), while the β-sheets provide the desirable nanoscale properties. Although previous work has established the efficacy of multidomain peptides, molecular-level characterization is lacking. The bioactive domains are intended to remain solvent-accessible without being incorporated into the β-sheet structure. We tested for three possible anticipated molecular-level consequences of introducing bioactive domains to β-sheet-forming peptides: (1) the bioactive domain has no effect on the self-assembling peptide structure; (2) the bioactive domain is incorporated into the β-sheet nanofiber; and (3) the bioactive domain interferes with self-assembly such that nanofibers are not formed. The peptides involved in this study incorporated self-assembling domains based on the (SL)_6_ motif and bioactive domains including a VEGF-A mimic (QK), an IGF-mimic (IGF-1c), and a *de novo* SARS-CoV-2 binding peptide (SBP3). We observed all three of the anticipated outcomes from our examination of peptides, illustrating the unintended structural effects that could adversely affect the desired biofunctionality and biomaterial properties of the resulting peptide hydrogel. This work is the first attempt to evaluate the structural effects of incorporating bioactive domains into a set of peptides unified by a similar self-assembling peptide domain. These structural insights reveal unmet challenges in the design of highly tunable bioactive self-assembling peptide hydrogels.

## Introduction

The ability to design multidomain peptides with a β-sheet-forming self-assembling peptide domain conjugated to a solvent-accessible bioactive domain is desirable in producing peptide hydrogels for biomedical applications.^1−4^ In a multidomain peptide, the self-assembling peptide domain promotes the formation of a nanofiber and a bioactive peptide domain is introduced to stimulate biological responses. Because of the modular nature of multidomain peptides, it may be tempting to assume that the structure and, consequently, function of a bioactive peptide mimic are retained despite incorporation into the self-assembling peptide. While some naturally occurring amyloids have flexible, solvent-exposed regions of the peptide that do not participate in the self-assembled nanofiber structure, we are aware of no existing reports about the structural consequences of constructing designer multidomain peptides with both self-assembling and bioactive domains.^5^ We hypothesize three major structural outcomes in incorporating bioactive peptide mimic to a self-assembling peptide: (1) the bioactive domain has no effect on the self-assembled nanofiber; (2) the bioactive domain is incorporated into the β-sheet, such that the nanofiber formation involves amino acids from both domains; and (3) the bioactive domain interferes with the self-assembly process, and nanofibers are not formed. We are aware of no current theory that can predict whether adding a bioactive domain will result in the proposed outcomes 1, 2, or 3.^6−10^

In this study, we used NMR as our primary technique to investigate whether the proposed structural outcomes could be detected from a system of peptides with and without bioactive domains. This work is the first to present molecular-level information about the self-assembled nanofiber formed by a set of multidomain peptides from combined solid- and solution-state NMR results. Table 1 lists all of the peptides studied in this article. We evaluated the differences in the nanofiber structure formed by the following self-assembling peptides conjugated with the bioactive peptide mimics: (1) the vascular endothelial growth factor (VEGF-A) bioactive mimic that provides a scaffold for angiogenesis,^6−8^ (2) the insulin-like growth factor (IGF-1) mimic that has several functions including downstream signaling and proliferation of somatic cells,^9−11^ and (3) SBP3, a mutated ACE2 receptor binding domain fragment, which binds (data not shown) to the spike protein of the SARS-Cov2-RBD-bound ACE2 complex.^12−14^ The efficacy of these systems from *in vivo* and *in vitro* studies, as well as the rational design of the bioactive peptide mimics, is reported elsewhere.^6,11,15^ By applying the one-dimensional (1D) solid-state NMR experiment ^1^H–^13^C cross-polarization magic angle spinning (CPMAS), we were able to identify the amino acids involved in the nanofiber structure and evaluate the structural order of the peptide samples. We discovered that all three of the hypothesized structural outcomes are possible. We also present additional results from modifications made to the polyglycine linker domain and producing stoichiometric mixtures of peptides. Prior to this work, the structural effects of the self-assembled nanofiber upon the incorporation of a bioactive domain have not been explicitly studied at the molecular level. A fundamental understanding of how structural design features may impact self-assembly and mimic presentation will be critical to the design and subsequent translation of next-generation *bioactive* self-assembling peptide scaffolds.

**Table 1:** Peptide Sequences and Molecular Weights

peptide	self-assembling domain	linker	bioactive domain	molecular weight (Da)
SL	K-SLSLSLSLSLSL-K			1516.82
SLan	K-SLSLSLSLSLSL-K	G	KLTWQELYQLKYKGI	3467.12
SLG_1_IGF	K-SLSLSLSLSLSL-K	G	GYGSSSRRAPQT	2822.18
SLG_2_IGF	K-SLSLSLSLSLSL-K	GG	GYGSSSRRAPQT	2879.23
SLG_3_IGF	K-SLSLSLSLSLSL-K	GGG	GYGSSSRRAPQT	2936.28
SLG_4_IGF	K-SLSLSLSLSLSL-K	GGGG	GYGSSSRRAPQT	2993.33
SLG_5_IGF	K-SLSLSLSLSLSL-K	GGGGG	GYGSSSRRAPQT	3050.38
SLG_6_IGF	K-SLSLSLSLSLSL-K	GGGGGG	GYGSSSRRAPQT	3107.44
E1	E-SLSLSLSLSLSL-E			1518.70
ESBP3	E-SLSLSLSLSLSL-E	G	QYKTYIDKNNHYAEDERYK	3995.37

## Materials and Methods

### Peptide Synthesis

All peptides (excluding E1 and ESBP3) were synthesized with standard Fmoc chemistry using a CEM Liberty Blue solid phase peptide synthesizer. Amino acids were dissolved in dimethylformamide (DMF) and conjugated to a Rink amide ProTide resin (0.18 mmol/g loading) at 90–95 °C. Peptides were acetylated and amidated at the C-terminus and N-terminus, respectively, then cleaved using a solution of 0.25 mL of Milli-Q water, 0.25 mL of 3,6-dioxa-1,8-octanedithiol (DODT), 0.25 mL of triisopropylsilane (TIS), and 9.25 mL of trifluoroacetic acid (TFA) for 30 min in a 37 °C water bath. After repeated cold ether washes, vortexing, and centrifugation, the 0.45 μm filtered crude peptide was obtained. The peptide was then dried overnight, resuspended, and prepared at a 1 mg/mL concentration in Milli-Q water with pH adjustments. Finally, the peptide was filtered through a 0.22 μm PTFE membrane and dialyzed in a 1000 Da cutoff dialysis tubing for 3 days. Peptides were frozen at −80 °C and lyophilized to a peptide powder that was stored at −80 °C prior to use for experiments. Peptide identities and molecular weights were verified using mass spectrometry conducted at the Georgia Institute of Technology Systems Mass Spectrometry Core (SyMS-C) facility. E1 and ESBP3 peptides were purchased from AmbioPharm, Inc. Mass spectra are presented in Figures S3–S12.

### Hydrogel Preparation

After peptide production, samples were prepared by dissolving peptides in buffer solutions. SL and SLan peptide solutions were prepared by dissolving 10 mg of peptide in 900 μL of Milli-Q water, vortexing the solution, and then adding 100 μL of 10× PBS to achieve the final concentration of 10 mg/mL of peptide in 1× PBS. IGF peptide solutions (SLG_1_IGF, SLG_2_IGF, SLG_3_IGF, SLG_4_IGF, SLG_5_IGF, SLG_6_IGF) were prepared by weighing approximately 10 mg of each peptide, dissolving them in 900 μL of Milli-Q water, vortexing, and titrating them with 1 M acetic acid and 1 M NaOH to achieve a pH of 7. 10× PBS was added to achieve a final concentration of 3.54 mM. E1, ESBP3, and 3:1 molar ratio E1: ESBP3 peptide solutions were prepared in 0.9% saline solution at the following concentrations: peptide solutions in 0.9% saline solution: 2.54 mM E1, 6.58 mM ESBP3, and a solution containing 2.54 mM E1 and 6.58 mM ESBP3 (3:1 molar ratio, total peptide concentration 3.55 mM). After dissolution, all peptide samples were incubated at room temperature on the benchtop overnight prior to solid-state NMR rotor packing.

### Solid-State Nuclear Magnetic Resonance (NMR) Spectroscopy

At least 10 mg of peptide is required to achieve the appropriate signal-to-noise ratio for NMR measurements. Peptide hydrogel samples were diluted 10-fold from approximately 10 to 1 mg/mL. Solutions were then ultracentrifuged for 30 min into Bruker 3.2 mm NMR rotors at 4 °C and 150,000 RCF with ultraclear tubes in an SW-41 Ti swinging bucket rotor fitted onto a Beckman Optima XPN-100 centrifuge. ^13^C cross-polarization magic angle spinning (CPMAS) spectra were collected on an 11.75 T magnet (500 MHz, ^1^H NMR frequency) with a 3.2 mm Bruker Low-E ^1^H/^13^C/^15^N NMR probe in a Bruker spectrometer. A 10 kHz magic angle spinning speed was used for all samples. Signals were averaged over approximately 12 h or 12,000 scans.

### Solution-State Nuclear Magnetic Resonance (NMR) Spectroscopy

Peptide samples were dissolved in deuterium oxide (D_2_O) at a concentration of 1 mg/mL prior to solution NMR measurements on an 11.75 T magnet (500 MHz, ^1^H NMR frequency) in a Bruker spectrometer. 1D ^1^H spectra were collected using the Bruker default *zgesgp* pulse sequence for solvent suppression using excitation sculpting at room temperature. Signals were averaged over 100 scans or approximately 10 min of scanning. 1D ^1^H Carr–Purcell–Meiboom–Gill (CPMG) solution NMR experiments were collected using the Bruker pulse sequence *cpmgesgp2d.* The recycle delay was set to 5 s, the CPMG time (d31) was set to 0.002 s, and shorter-to-longer CPMG (T2) relaxation filter values were 5, 25, 50, and 800. Signals were averaged over 64 scans.

### Transmission Electron Microscopy (TEM)

Solutions of 1 mg/mL E1 and 3:1 E1/ESBP3 combination peptide samples were prepared in water by first measuring the peptide gravimetrically and then adding it to the appropriate amount of water. ultraviolet/visible (UV/vis) absorption was used to confirm the peptide concentrations. After peptide solutions were allowed to sit for 1 h, TEM grids were prepared using 400-mesh lacey, carbon-coated, copper grids (Ted Pella, Inc.). For each peptide solution, a 5 μL drop was spotted on the TEM grid and left for 2 min. The drop containing the peptide solution was then blotted away with filter paper. Next, a 5 μL drop of water was spotted on the grid and blotted away after 1 min. Spotting with 5 μL of peptide solution and water was repeated 2 more times. Finally, a 5 μL drop of 1 wt % uranyl acetate was blotted away after 1 min, and grids were left to air-dry. Transmission electron micrographs were acquired with a 120 kV JEOL JEM1400 on a 4k × 4k Gatan UltraScan 1000 CCD camera (Gatan) using 80 kV for measurements. A solution of ESBP3 at 10 μM was stained with 1% (v/v) 430 ammonium molybdate (in water) for 2 min, then washed with water, and air-dried. Transmission electron micrographs of ESBP3 were collected on a JEOL 2200FS electron microscope.

### Thioflavin T (ThT) Fluorescence

E1, ESBP3, and 3:1 molar ratio E1/ESBP3 peptide samples were prepared at a concentration of 2.54 mM in 0.9% saline solution prior to the addition of 0.08 mg/mL ThT and 1× PBS. A BioTek Synergy H4Microplate Reader was used to measure the intensity of solutions pipetted into a black 96-well plate (Thermo Scientific Nunc) at excitation and emission wavelengths of 450 and 482 nm, respectively. Samples were run in triplicate over 48 h of measurement. The average fluorescence intensity is reported in the main text.

### Rheology

The rheological characterization of the peptide hydrogels was performed using a TA Instruments HR-2 Discovery Hybrid Rheometer equipped with a 40 mm diameter parallel plate spindle. Approximately 300 μL of the peptide hydrogel was deposited onto the sample stage, ensuring complete coverage of the plate area as the spindle approached the substrate. A TA Instruments DHR Solvent Trap was used to prevent water evaporation during the shear rheology experiments. Oscillatory shear strains ranging from 0.1 to 100% were applied at a frequency of 1 Hz. The storage modulus and loss modulus were recorded as functions of the shear strain amplitude. Measurements were conducted at 20 °C and performed in triplicate.

## Results and Discussion

**Figure 1:**
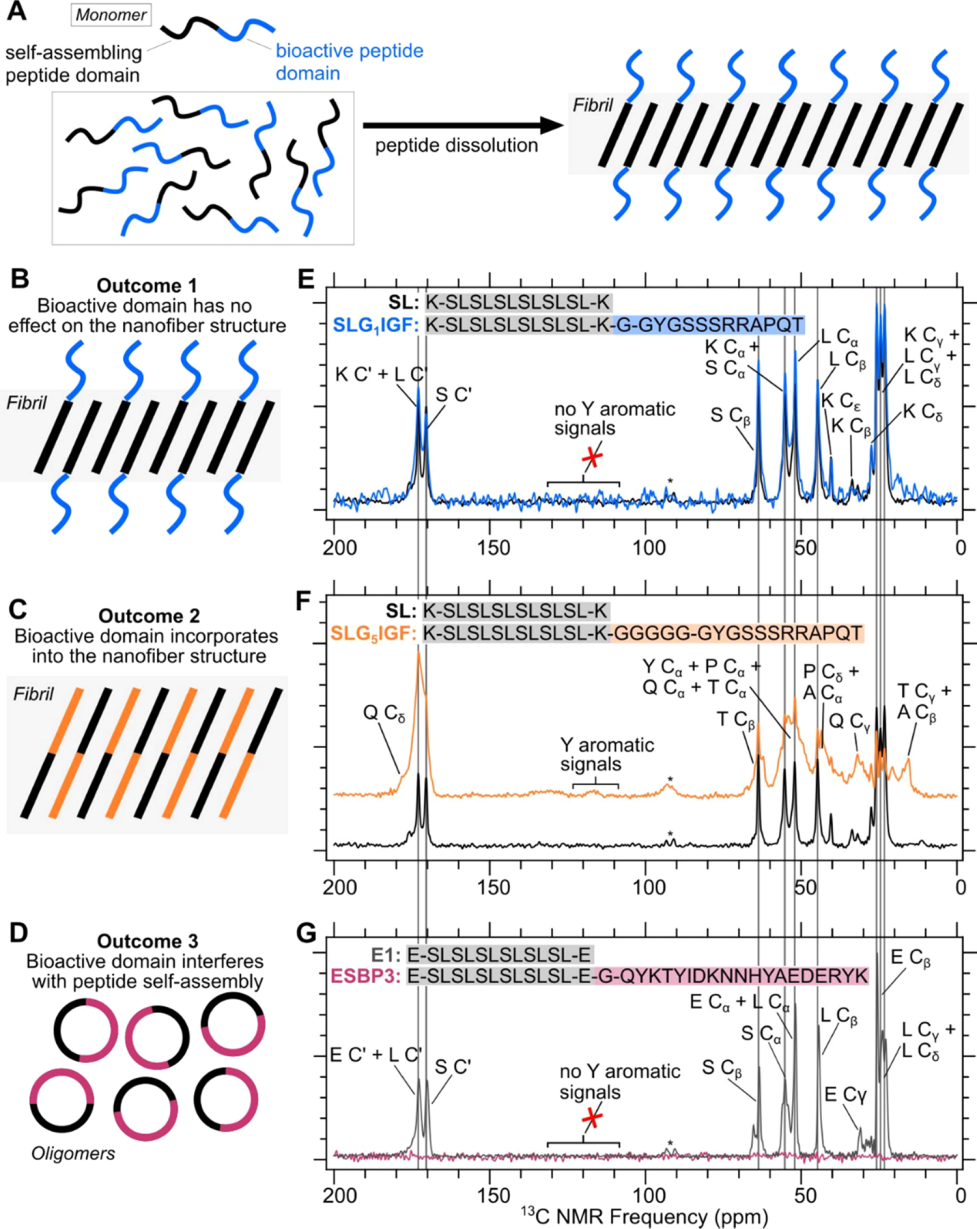
Cartoons and NMR spectra summarizing the three possible structural outcomes of incorporating bioactive peptide domains into self-assembling peptide domains. In each cartoon, the self-assembling peptide domain is represented by a black line. The bioactive domain is represented by colored lines. (A) Diagram illustrating the peptide self-assembly process. (B) Cartoon depiction of outcome 1: bioactive domain has no effect on the self-assembled structure. (C) Cartoon depiction of outcome 2: bioactive domain incorporates into the self-assembled structure. (D) Cartoon depiction of outcome 3: bioactive domain interferes with the self-assembled structure. (E) Overlay of SL and SLG_1_IGF spectra. (F) Stacked plot of SL and SLG_5_IGF spectra. (G) Overlay of E1 and ESBP3 spectra. NMR peaks are labeled according to the known β-shift chemical ranges for carbon atoms within the residues. Gray vertical lines align peaks from S and L amino acids from the SL and E1 self-assembling peptides. Asterisks (*) indicate peaks from spinning side bands caused by magic angle spinning.

This work is the first report that uses solid-state NMR to survey a set of 10 multidomain peptides and evaluate whether the structure of the self-assembled peptide nanofiber is altered after the addition of a bioactive peptide mimic. A diagram in Figure 1A shows the general process of nanofiber formation after multidomain peptides are dissolved in water or buffer. We hypothesize three possible structural outcomes for peptides with self-assembling and bioactive domains and summarize them using illustrations in Figure 1B–D. Outcome 1, considered the desired scenario for incorporating bioactive domains, occurs when the bioactive domain has no impact on the self-assembled structure (Figure 1A). In this case, the bioactive domain can remain solvent-accessible and retain its biological functionality. A cartoon depiction of outcome 1 assumes an antiparallel β-strand alignment between self-assembling peptides across the β-sheet nanofiber for simplicity, where the black and blue lines represent the self-assembling peptide and the bioactive peptide domain, respectively. The cartoon depiction also assumes that the bioactive domain maintains a uniform structure across the nanofiber, although this is not proven. The actual structure of the bioactive domain can be derived based on molecular structures of the initial protein complexes determined by cryo-EM and X-ray crystallography. The second possible outcome is when the bioactive domain incorporates itself into the nanofiber structure (Figure 1B). In contrast to outcome 1, the incorporation of the bioactive domain does not allow it to remain solvent-accessible or to retain its original structure. A likely result is that the biological functionality of the bioactive peptide mimic is reduced. For simplicity, the cartoon in Figure 1B assumes that the incorporation of the bioactive peptide is uniform across the fibril. The third hypothesized outcome occurs when there is no nanofiber formation present. The cartoon shown in Figure 1C outlines the possibility of oligomer formation as opposed to fiber formation. Another possibility is that the multidomain peptides are nonstructural, and the peptide solution is composed of dynamic and random coil monomeric peptides.

^1^H–^13^C cross-polarization magic angle spinning (CPMAS) is the main technique applied to assess the sample structural order and identify the amino acids involved in the self-assembled β-sheet peptide nanofiber. The ^13^C signals from CPMAS will aid in identifying the amino acids directly involved in the rigid (nondynamic) regions, such as the self-assembled peptide nanofiber.^16,17^ Amino acids located in dynamic and solvent-accessible regions of the peptide are not anticipated to show measurable cross-polarization effects and will not produce detectable ^13^C signals. Partial peak ^13^C assignments can be made based on the known chemical shift statistics of carbon atoms within each amino acid.^18−22^ While experiments conducted with isotope labeling can provide more detailed information about the precise atomic arrangement, labels are not necessary for detecting and distinguishing between the three hypothesized structural outcomes presented above. All 10 peptides examined in this study were synthesized without the incorporation of isotopes. This approach allowed us to test possible structural outcomes on a larger set of peptides versus the conventional structural biology approach of running NMR measurements on a single peptide with different isotope labels. Information gathered from solution NMR, electron microscopy, thioflavin T fluorescence, and rheology also assisted in evaluating the peptides.

A comparison of CPMAS measurements on SL and SLG_1_IGF showed the experimental possibility of outcome 1, the desired result in designing biofunctional multidomain peptides (Figure 1E). The spectra produced from the two peptides produced similar spectra, with chemical shifts corresponding to the carbon atoms within the K, S, and L residues. Virtually no ^13^C signals are detected from the residues that comprise the bioactive peptide domain. No aromatic signals are observed in the spectrum of SLG_1_IGF, indicating that the residue Y from the bioactive domain is unlikely to participate in the nanofiber structure.^21^ Peak line widths (full width at half-maximum) from the two spectra are between 1 and 2 ppm, demonstrating that the samples produced highly ordered assemblies typical of those observed from amyloid.^23,24^ Broad peak line widths in the CPMAS spectra (>2 ppm) are from the spectral overlap of peaks from multiple amino acids with similar chemical shifts. The spectral overlay also shows that SL has a higher signal-to-noise than SLG_1_IGF. Both SL and SLG_1_IGF were prepared at 10 mg/mL of peptide concentration or 6.59 and 3.54 mM, respectively. Both samples were also scanned for 12 h or 12,000 scans. After scaling the results according to the noise level and integrating the carbonyl carbon peaks from both samples, we determined that SLG_1_IGF has approximately 55% of the overall signal of SL (see Figure S1). This difference in the overall signal is proportional to the molar concentration of the SL sample compared to that of the SLG_1_IGF sample. Signal-to-noise also depends on the overall amount of material in the rotor, the spectral line widths, and molecular dynamics. Peptide samples can produce varying amounts of peptide in the centrifuged pellet depending on the degree of peptide self-assembly. The difference in signal-to-noise is not reflective of any difference in structural distributions within each sample. Altogether, the NMR spectra in Figure 1E suggest that the multidomain peptide SLG_1_IGF has a nanofiber formed only by the residues within the self-assembling peptide domain. The incorporation of the IGF-1c peptide mimic into SL has no effect on the self-assembled structure.

The NMR results of SLG_5_IGF show that the IGF-1c peptide mimic incorporates itself into the nanofiber structure, confirming the experimental possibility for outcome 2 (Figure 1F). Stacked spectra of SL and SLG_5_IGF are presented, with gray lines drawn to indicate chemical shifts from the carbons within the S and L amino acids. Additional peaks appear in the SLG_5_IGF spectrum that do not align with the S and L chemical shifts, indicating that amino acids beyond S and L were involved in the self-assembled nanofiber formation. Partial peak assignments reveal the presence of carbon signals from Q, Y, P, Q, T, and A. Additionally, weak aromatic signals are detected in the aromatic region (∼110–140 ppm) of the spectrum.^21,22^ Broadening of the peaks in the SLG_5_IGF spectrum is due to overlapping carbon signals from multiple residues. From outcome 2, we suggest that the incorporation of additional amino acids from the bioactive domain into the self-assembled nanofiber impedes the bioactive domain’s ability to maintain its original structure and interact with the environment.

Outcome 3 was detected in another system of peptides, E1 and ESBP3. Although E1 and ESBP3 peptide solutions were prepared in the same conditions (10 mg/mL peptide concentration in 0.9% saline buffer and ultracentrifugation into NMR rotors for 30 min), CPMAS measurements only detected the formation of self-assembled nanofibers in the E1 sample. Figure 1G shows a comparison of the E1 and ESBP3 spectra. We observe ^13^C signals corresponding to the E, S, and L residues and narrow peak line widths (∼1–2 ppm) for the E1 sample, consistent with an ordered assembly. In contrast, ESBP3 does not show a measurable CPMAS signal above the noise. The addition of the SBP3 bioactive domain to the E1 self-assembling peptide domain disrupted the formation process of a self-assembled nanofiber and did not produce any detectable NMR signals from CPMAS. Experiments described in a later section confirm the presence of smaller nonfibrillar aggregates formed by ESBP3 that are not detectable by CPMAS measurements. Interestingly, from the set of peptide systems studied in this work, we were able to detect all three hypothesized structural outcomes.

**Figure 2:**
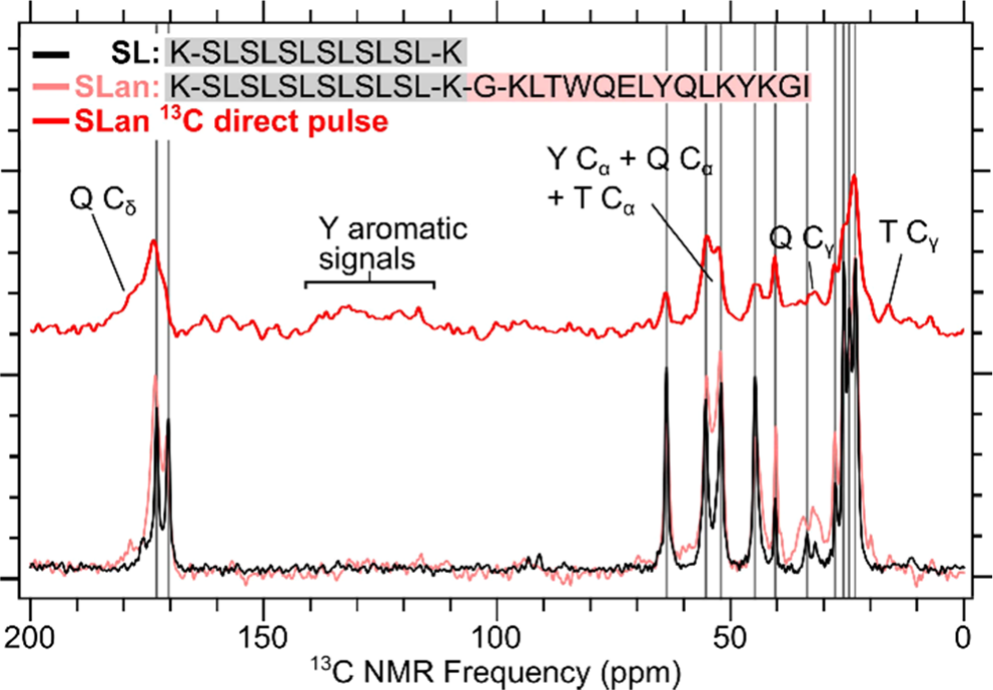
1D ^13^C direct pulse spectrum of SLan and ^1^H–^13^C CPMAS measurements of SL and SLan. Gray vertical lines indicate peaks from the S, L, and K amino acids. Black arrows identify additional ^13^C peaks from the direct pulse measurement of SLan.

Another one-dimensional ^13^C solid-state NMR technique, ^13^C direct pulse NMR, can be used to detect ^13^C signals from amino acids that do not participate in the rigid, self-assembled nanofiber.^25,26^ CPMAS measurements conducted on the peptides SLan and SL produced results consistent with outcome 1 (Figure 2). We observed similar ^1^H–^13^C CPMAS spectra between SLan and SL upon comparison. Primarily, K, S, and L signals appear, suggesting that these three amino acids participate in the self-assembled nanofiber. Direct pulse measurements on SLan reveal the remaining ^13^C signals from the amino acids in the bioactive peptide mimic VEGF-165. The aromatic region, between 110 and 140 ppm, shows signals from the amino acid Y present in the bioactive domain. Signals below 20 ppm can be assigned to the amino acid T. Signals above 180 ppm are from the carbonyl side chain of E. Peaks at around 160 ppm are from Q.^21,22^ The results from direct pulse measurements confirm that although we do not see all signals in our ^1^H–^13^C CPMAS measurement, we can still verify the presence of the mobile residues in the bioactive domain by using other NMR techniques.

**Figure 3:**
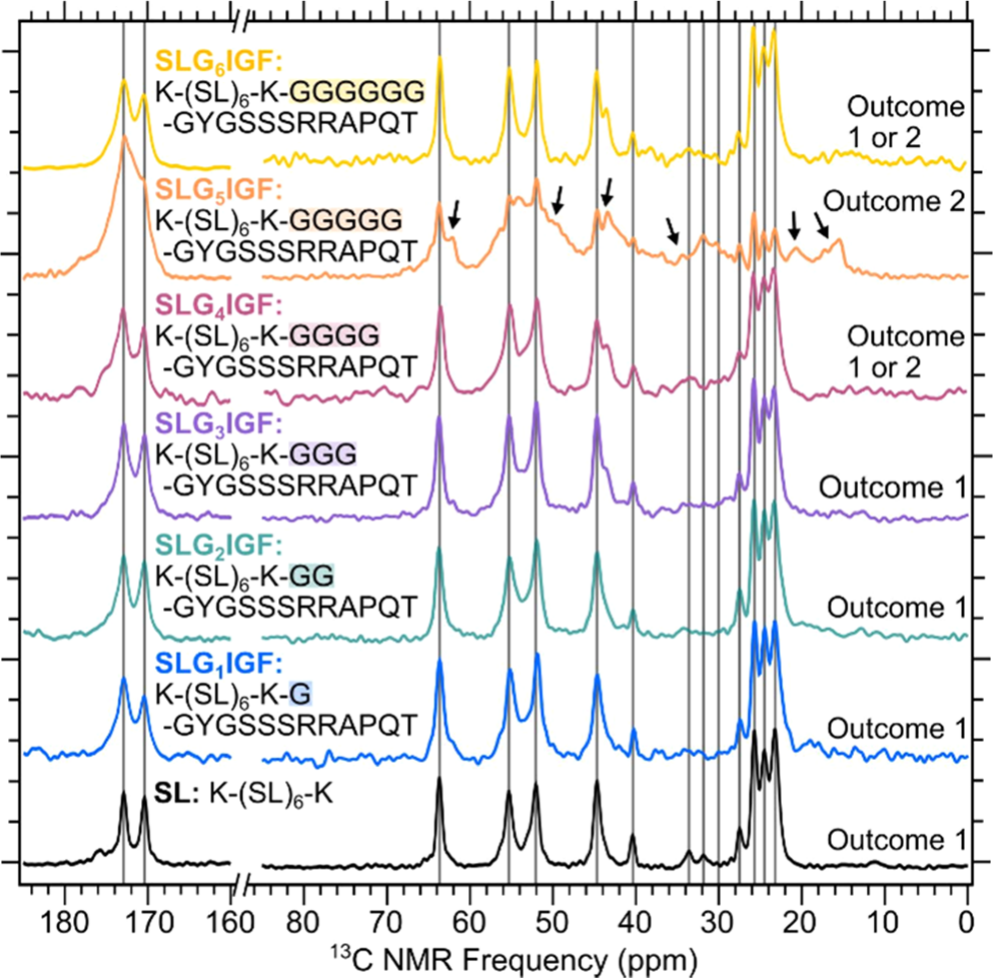
^1^H–^13^C CPMAS measurements for the IGF series of peptides (SL, SLG_1_IGF, SLG_2_IGF, SLG_3_IGF, SLG_4_IGF, SLG_5_IGF, and SLG_6_IGF). Black arrows indicate additional peaks in the SLG_5_IGF spectra that are not present in the SL spectrum. Gray vertical lines indicate peaks from the S, L, and K residues.

To investigate whether multidomain peptides are sensitive to changes in the peptide linker domain connecting the self-assembling peptide domain to the bioactive domain, we collected CPMAS measurements on a series of IGF peptides. Figure 3 shows a stacked plot of CPMAS measurements from a set of six IGF peptides that contain the self-assembling peptide SL, linked by one to six glycine residues to the bioactive peptide mimic IGF-1c. Gray lines in Figure 3 show signals from the SL peptide (partial ^13^C assignments shown in Figure 1A) that correspond to K, S, and L amino acids. SLG_1_IGF, SLG_2_IGF, and SLG_3_IGF have spectra similar to those of SL, with all peaks aligning with the signals from K, S, and L. SLG_4_IGF and SLG_6_IGF show spectra with behavior between outcome 1 or 2. SLG_5_IGF produced a spectrum that reflects outcome 2, where additional ^13^C signals are present from amino acids within the bioactive mobile region of the peptide. All samples produced NMR spectra with narrow peak line widths (∼1–2 ppm) typical of an ordered assembly. The broad peaks present in SLG_4_IGF, SLG_5_IGF, and SLG_6_IGF are due to overlapping signals from multiple residues. The transition between outcomes 1 and 2 from the addition or deletion of glycine shows the sensitivity of the amino acid sequence in the nanofiber formation process.

**Figure 4:**
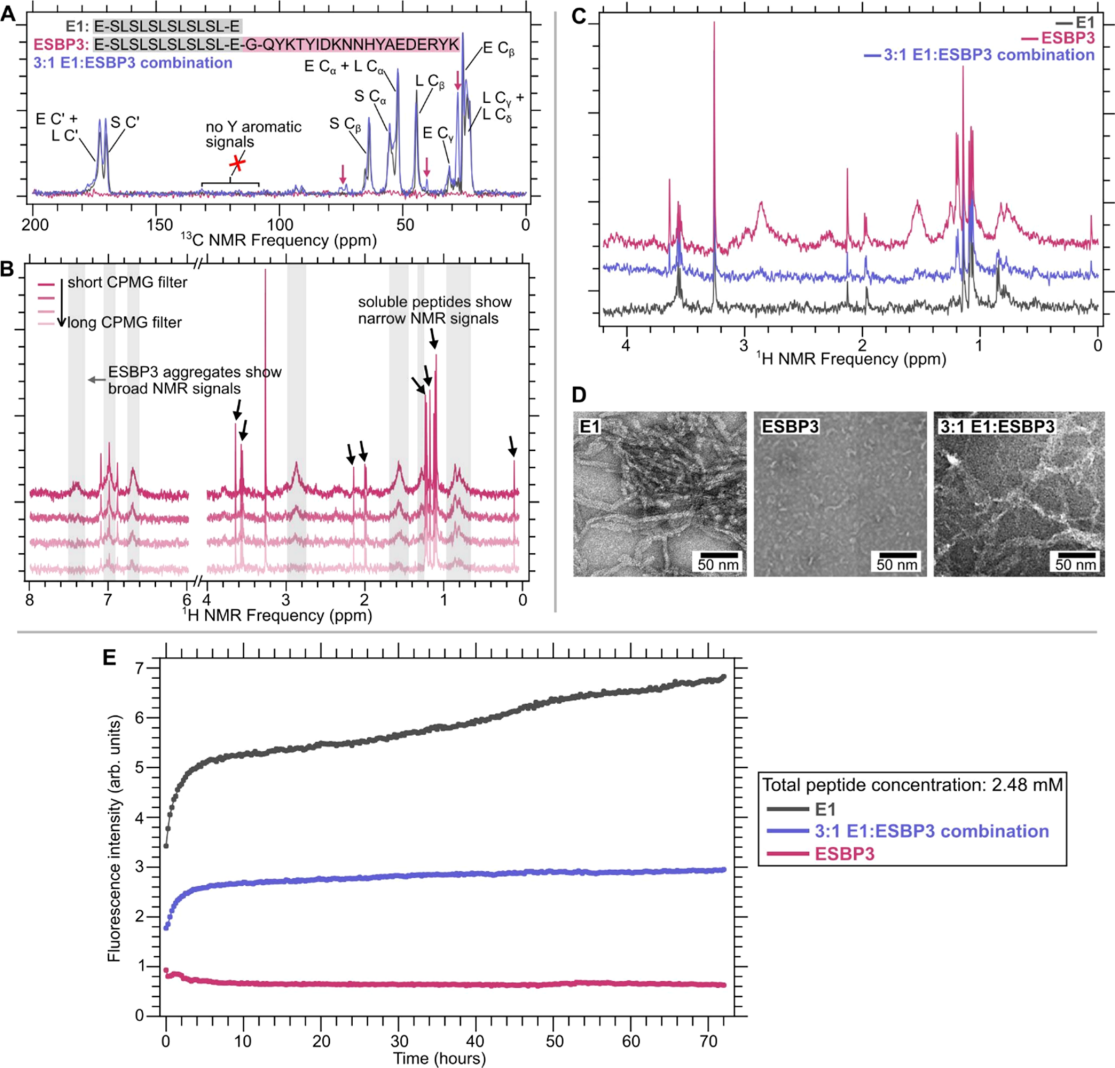
Solid-state NMR, solution-state NMR measurements, and TEM images for E1, ESBP3, and 3:1 E1: ESBP3 combination. (A) ^1^H–^13^C CPMAS spectra comparing E1, ESBP3, and 3:1 ESBP3. Labeled peaks are from partial assignments to carbon atoms in residues by using the known chemical shift ranges for β-sheets. The pink arrow shows an additional signal present in the 3:1 E1/ESBP3 sample that is not present in the other two spectra. (B) CPMG measurements on ESBP3. Black arrows indicate narrow lines that are from the soluble, monomeric peptides. Gray regions highlight broad NMR signals from soluble aggregates that change as the CPMG filter changes. (C) ^1^H solution NMR spectra of the three samples highlighting changes in free, monomeric peptides and soluble aggregate species. (D) TEM images of the three samples. TEM image for ESBP3 is reprinted (no changes) from Dodd-o et al., *Nat Commun***15**, 1142 (2024),^15^ under the Creative Commons CC license (http://creativecommons.org/licenses/by/4.0/). (E) Average thioflavin T fluorescence intensity for the three samples.

We hypothesize that we can recover the undesirable result of outcome 3 by “co-assembling” peptides with and without the bioactive domain. Similar strategies to combine a peptide with a variant of the same peptide have been used to create biomaterials.^27,^ To test if this technique can be applied to the E1 and ESBP3 systems, we prepared a sample of a 3:1 molar ratio E1/ESBP3. Figure 4A shows a recovery of NMR signals from the 3:1 E1/ESBP3 sample with good alignment to NMR signals from the E1 spectrum. An overlay of E1 and 3:1 ESBP3 spectra exhibits peak line widths of ∼1–2 ppm, which is typical for ordered assemblies. Broad peaks are from overlapping ^13^C signals. Because E1 is the most abundant peptide in the E1 and 3:1 E1/ESBP3 samples, most peaks can be assigned to E, S, and L ^13^C signals.^21,22^ The 3:1 E1/ESBP3 spectrum also shows a unique signal at 28 ppm that was not present in the E1 spectrum, suggesting that the addition of ESBP3 to E1 alters the self-assembled nanofiber structure. Residues from the SBP3 domain are likely incorporated into the β-sheet assembly, producing a structural outcome closer to that of outcome 2.

^1^H and Carr–Purcell–Meiboom–Gill (CPMG) solution-state NMR spectra were conducted to test whether there was any evidence of peptide assembly from soluble aggregates, which was implied by the loss of signal from solid-state NMR.^29,30^Figure 4B shows the results from ^1^H and CPMG experiments on the ESBP3 sample. We observed both sharp and broad NMR signals from the soluble species. Sharp peaks with narrow line widths (<0.1 ppm full width at half-maximum) are typical of free, monomeric peptides in solution. Broad solution NMR peaks (∼0.2 ppm full width at half-maximum) in the ESBP3 spectra indicate the presence of soluble nonfibrillar aggregates.^31^ By using optimized pulses in the CPMG pulse program, we filtered out NMR signals that correspond to larger molecules. The loss of the NMR signal to the broad peaks using longer CPMG filters confirmed the presence of soluble nonfibrillar aggregates. Figure 4C shows ^1^H solution-state NMR spectra containing a limited number of sharp signals for the E1, ESBP3, and 3:1 E1/ESBP3 combination samples, which is consistent with the formation of both monomeric and self-assembled nanofiber structures. In the case of the 3:1 E1/ESBP3 combination sample, the addition of 3-parts E1 to 1-part ESBP3 eliminated the broad signals present in the ESBP3 sample alone. We believe that the soluble aggregates present in the ESBP3 sample are eliminated after the addition of E1. There are two possible phenomena that can explain this observation: (1) E1 self-assembles and “cross-seeds” ESBP3 assembly, where samples comprise individual fibrils of only E1 peptides and fibrils of only ESBP3 peptides, and (3) E1 and ESBP3 “coassemble” such that both peptides are found in every fibril. From our solution-state NMR data, both phenomena are possible. However, we cannot differentiate between the two without further solid-state NMR measurements on samples with isotopic labels.

TEM imaging and thioflavin T (ThT) fluorescence were also conducted to differentiate among the samples. Figure 4D shows TEM images of the three samples that are consistent with the solid-state and solution NMR data of the samples. E1 and 3:1 E1/ESBP3 show the formation of fibrils, while ESBP3 shows smaller aggregates. The changes in morphology observed in TEM suggest a change in the structure between the samples. ThT measurements show an increase in fluorescence intensity for E1 and 3:1 E1/ESBP3, with a higher overall intensity detected for E1 (Figure 4E). An increase in fluorescence intensity occurs as ThT molecules bind to β-sheets over time.^32^ ESBP3 did not show any noticeable increase in the ThT signal. Altogether, the biophysical measurements on the E1, 3:1 E1/ESBP3 combination, and ESBP3 samples further validate the results from CPMAS measurements that the E1 and 3:1 E1/ESBP3 samples produce fibrils, while ESBP3 does not. We also show that although ESBP3 does not form fibrils, smaller aggregates are present in the peptide solution.

**Figure 5:**
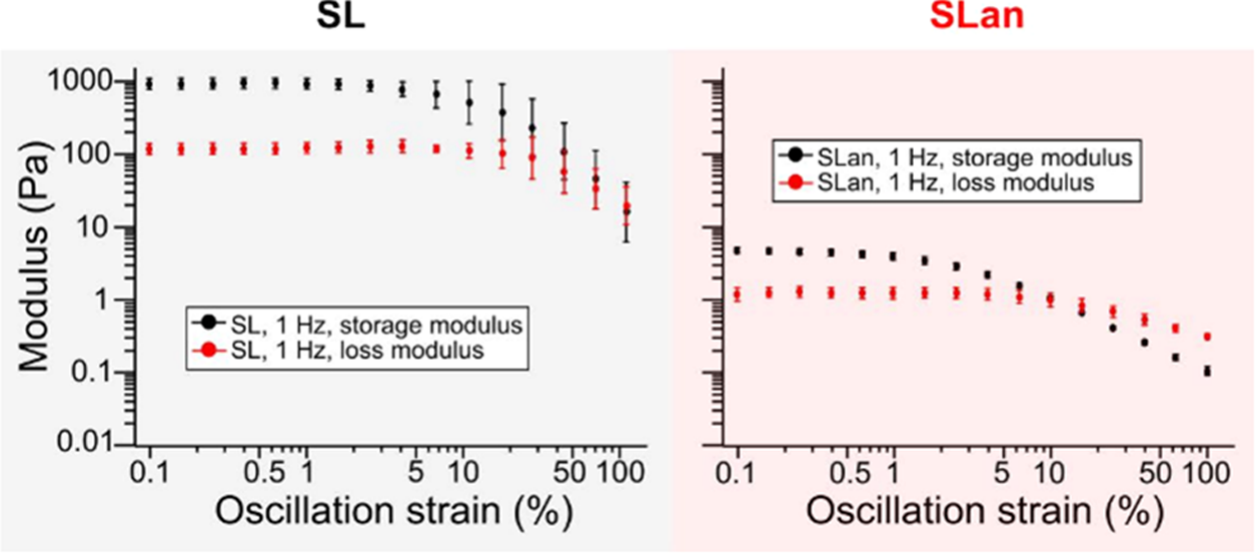
Shear rheology tests of SL (A) and SLan (B) peptide hydrogels subject to 0.1–100% strain at a 1 Hz frequency of oscillation. In each panel, the black markers show the storage modulus, and the red markers show the loss modulus as a function of the oscillation strain. Error bars indicate a 95% confidence interval.

Even though the two peptides produce the same CPMAS spectrum (outcome 1), the mechanical properties of the peptide hydrogels may differ. The mechanical properties of the peptide hydrogels depend on the structural properties of the polymer network, while the CPMAS spectrum provides information about the structural information on the β-sheets and not the network properties. To test this, we conducted shear rheological analysis on peptides SL and SLan. Figure 5 shows that for both hydrogels, the storage modulus is bigger than the loss modulus at small strains, meaning that the hydrogels behave more solid-like. As the shear strain increases, the storage modulus starts to decrease, and at a certain oscillation strain, the loss modulus starts to exceed the storage modulus. In other words, the hydrogels experience a solid–fluid transition process as the deformation increases. SLan was observed to have approximately 2 orders of magnitude lower shear storage modulus and loss modulus than SL, indicating that the effective cross-linking of the SLan polymer network is much less than the SL.^33−36^ Additionally, the SL peptide hydrogel showed a storage modulus greater than loss modulus up to about 50% oscillation strain, while the SLan peptide hydrogel experienced a transition point at a 10% oscillation strain, revealing that the SLan polymeric network is easier to disassemble than SL. A possible explanation is that additional interactions from the bioactive domain weaken the interactions between fibrils. As a result, the cross-linking of fibers within the SLan peptide hydrogel is more difficult to form and is easier to disconnect. Despite both peptide hydrogels exhibiting similar NMR spectra, the mechanical properties of the two hydrogels are significantly different. These results demonstrate that other design considerations may be important when designing peptide hydrogels for specific applications.

Altogether, the results from this study provide several insights into the structural complexity involved in designing multidomain peptides with biological functionality. While the heuristics-based design of alternating hydrophobic and hydrophilic amino acids is generally effective in promoting β-sheet formation, the final assembled structure does not always match design expectations.^3,,38^ Our experimental evidence shows that the nanofiber structure formed by self-assembling peptides can be impacted by the addition of biofunctional domains. In addition, the length and residues involved in the peptide linker domain can also affect the self-assembled structure. Our measurements on a series of IGF peptides linked by a polyglycine peptide domain demonstrate the sensitivity of the self-assembled structure to the amino acid sequence.

Further structural studies of peptides that incorporate other bioactive peptide domains can aid in understanding the key design considerations for multidomain peptides. In this work, we demonstrated the ability of CPMAS measurements to detect and distinguish between three structural outcomes for a set of self-assembling peptides and multidomain peptides with bioactive domains without the use of isotopic enrichment. NMR experiments on peptides with isotopic labels can provide the structural details necessary to build molecular models.^39^ Although previous literature has examined the role of polyglycine- and glycine-rich linkers to separate multiple domains in a single protein, we are aware of no other study that tests the structural impacts of linker length between a self-assembling peptide and a bioactive peptide domain.^40,41^ Structural studies on peptides with linker domains that use other residues can aid in determining optimal sequences that do not hamper the desired self-assembly behavior and biofunctionality. Recent efforts have been successful in the development of computational algorithms to design self-assembling peptides that form parallel and antiparallel β-sheet structures.^42,43^ These algorithms can also be adopted for the amino acid sequence design of multidomain peptides.

## Conclusions

Prior to this study, limited knowledge existed regarding the structural implications of introducing bioactive domains into self-assembled peptide structures. Through our solid- and solution-state NMR experiments, we confirmed the possibility of three distinct structural outcomes that may have consequences for bioactivity and biomaterial performance. The observed structural outcomes were proposed by listing different possibilities of how the bioactive domain can impact nanofiber formation. SLan and SLG_1_IGF are examples of peptides exhibiting the desired structural outcome 1, where the bioactive domain does not interfere with the self-assembled nanofiber structure. We also observed that residues from the bioactive domain can incorporate themselves into the self-assembled nanofiber in the study of SLG_5_IGF. In the case of ESBP3, the incorporation of the SBP3 bioactive peptide mimic interfered with the self-assembly altogether. Switching between different outcomes is also possible by manipulating the amino acid sequence, such as extending the peptide linker domain, or producing stoichiometric mixtures of peptides (*e.g*., coassembly). We currently lack a predictive mechanism to anticipate how the addition of a bioactive domain will affect the peptide self-assembly process. Further structural investigations on the effects of incorporating bioactive peptide domains into self-assembling peptide hydrogels can inform the design and translation of bioactive peptide-based materials for therapeutic applications.

## Supporting Information Available

Overlay of scaled SL and SLG_1_IGF CPMAS spectra; ThT replicate curves for E1, ESBP3, and 3:1 E1/ESBP3 samples; and mass spectra of SL, SLan, SLG_1_IGF, SLG_2_IGF, SLG_3_IGF, SLG_4_IGF, SLG_5_IGF, SLG_6_IGF, E1, and ESBP3 (PDF)

## Abbreviations

NMR: nuclear magnetic resonance
CPMAS: cross-polarization magic angle spinning
TEM: transmission electron microscopy
CPMG: Carr–Purcell–Meiboom–Gill
