# Supplementary material for: Structural Consequences of Introducing Bioactive Domains to Designer β-Sheet Peptide Self-Assemblies
Source: Biomacromolecules. 2024 Feb 26;25(3):1429–38. doi: 10.1021/acs.biomac.3c00962 (PMC10934295; doi:10.1021/acs.biomac.3c00962)
Supplement: Supplementary file 1 — bm3c00962_si_001.pdf [file bm3c00962_si_001.pdf]

Supplemental Information

Structural consequences of introducing bioactive domains to designer β-sheet peptide self-assemblies

Alicia S. Robang[1], Abhishek Roy[2], Joseph B. Dodd-o[2], Dongjing He[3], Justin V. Le[1], Andrew C. McShan[4], Yuhang Hu[1,3,5], Vivek A. Kumar[2,6,7], Anant K. Paravastu[1,5]

1. School of Chemical and Biomolecular Engineering, Georgia Institute of Technology, Atlanta, GA 30332, USA

2. Department of Biomedical Engineering, New Jersey Institute of Technology, Newark, NJ, 07102, USA

3. George W. Woodruff School of Mechanical Engineering, Georgia Institute of Technology, Atlanta, GA, 30332, USA

4. School of Chemistry and Biochemistry, Georgia Institute of Technology, Atlanta, GA 30332, USA

5. Parker H. Petit Institute for Bioengineering and Biosciences, Georgia Institute of Technology, Atlanta, GA 30332, USA

6. Department of Chemicals and Materials Engineering, New Jersey Institute of Technology, Newark, NJ, 07102, USA

7. Department of Biology, New Jersey Institute of Technology, Newark, NJ, 07102, USA

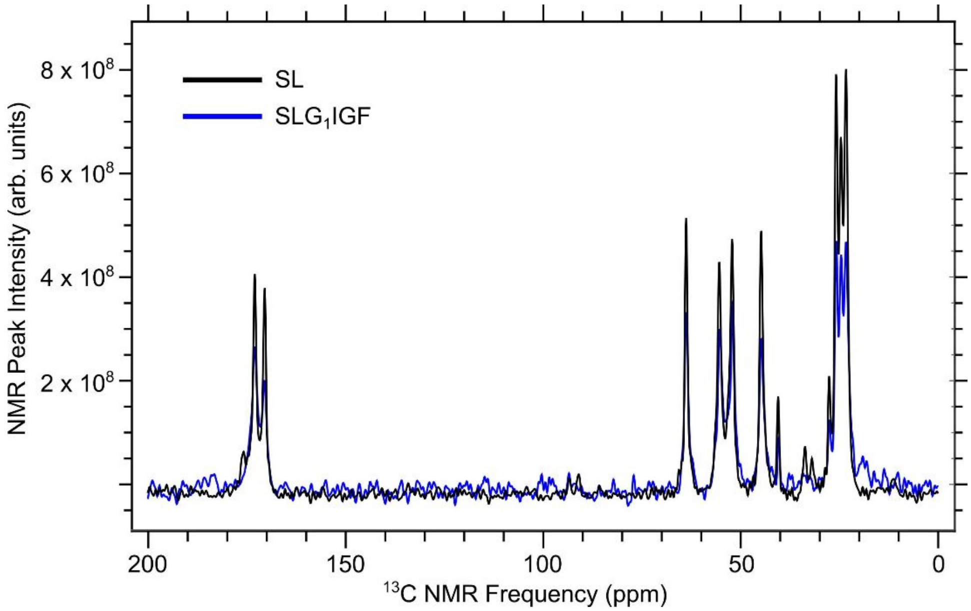

Supplemental Figure 1. Overlay of ^{1}H-^{13}C CPMAS measurements on SL and SLG_1IGF scaled so that the noise level is the same in both spectra.

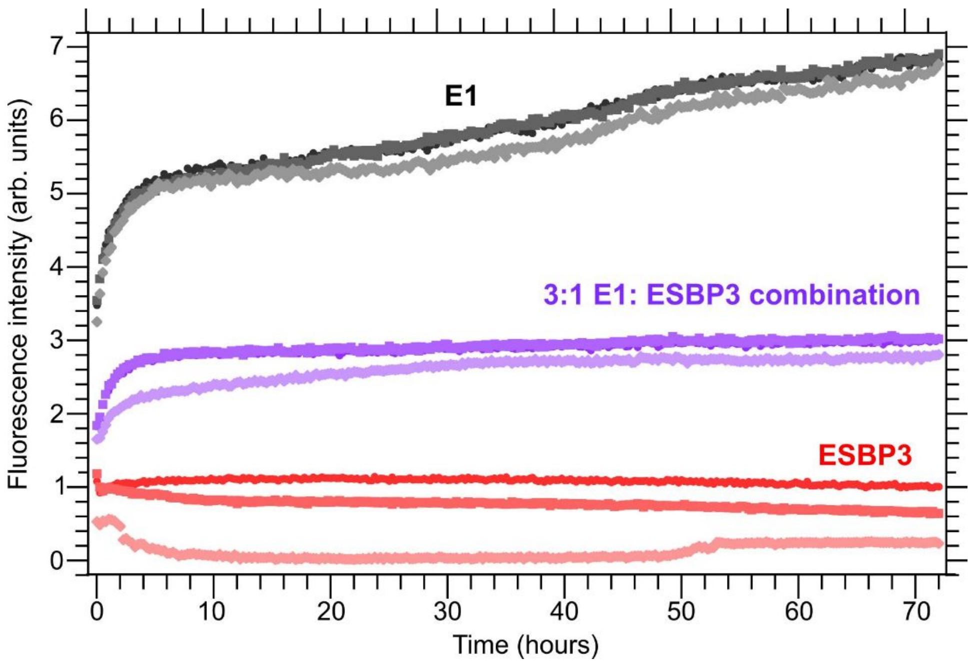

Supplemental Figure 2. Thioflavin T fluorescence replicate curves for E1, ESBP3, and 3:1 E1:ESBP3 combination samples, each prepared at a total peptide concentration of 2.48 mM.

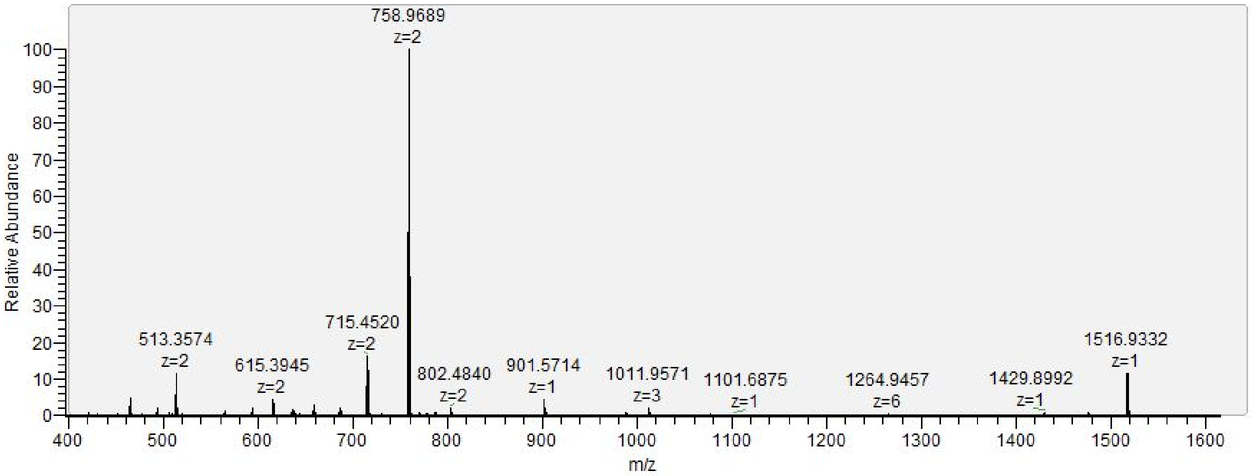

Supplemental Figure 3. Mass spectrum of SL.

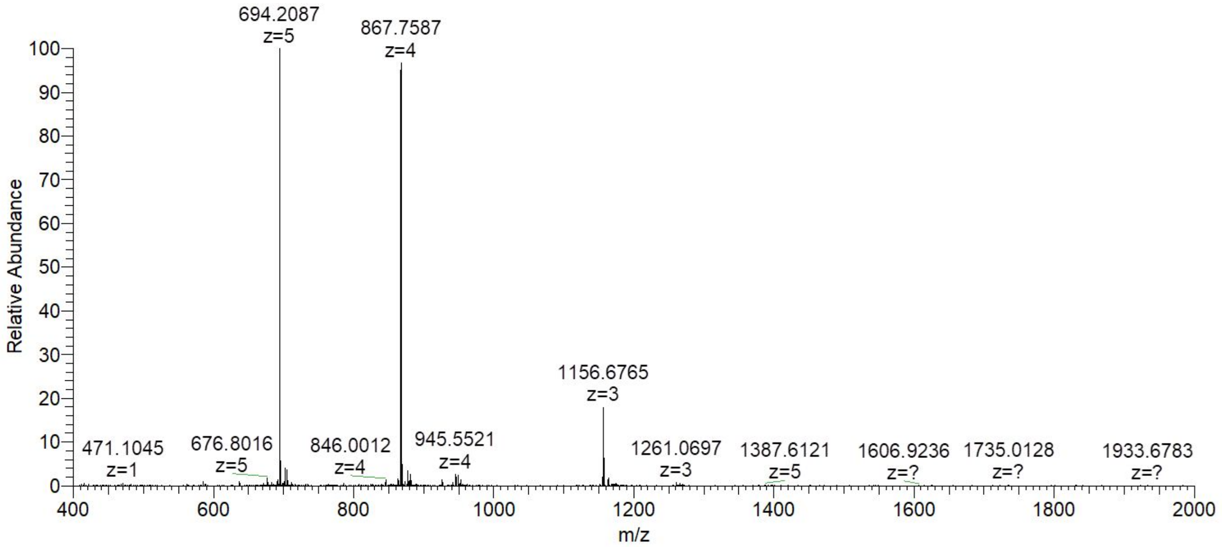

Supplemental Figure 4. Mass spectrum of SLan.

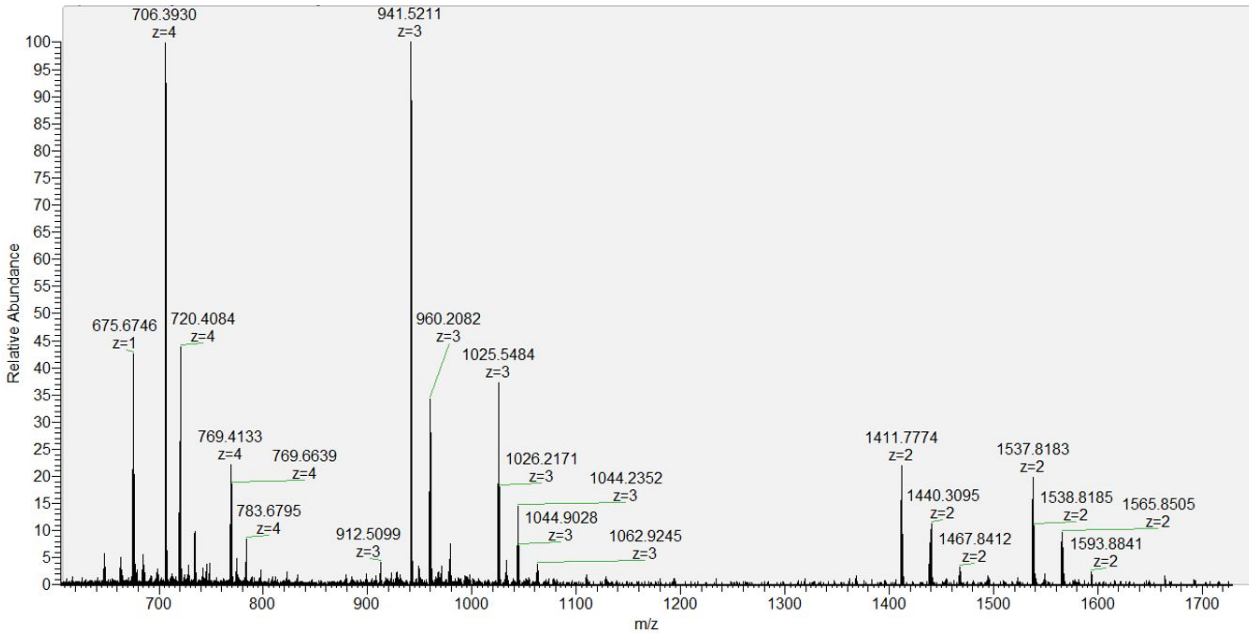

Supplemental Figure 5. Mass spectrum of SLG_1IGF.

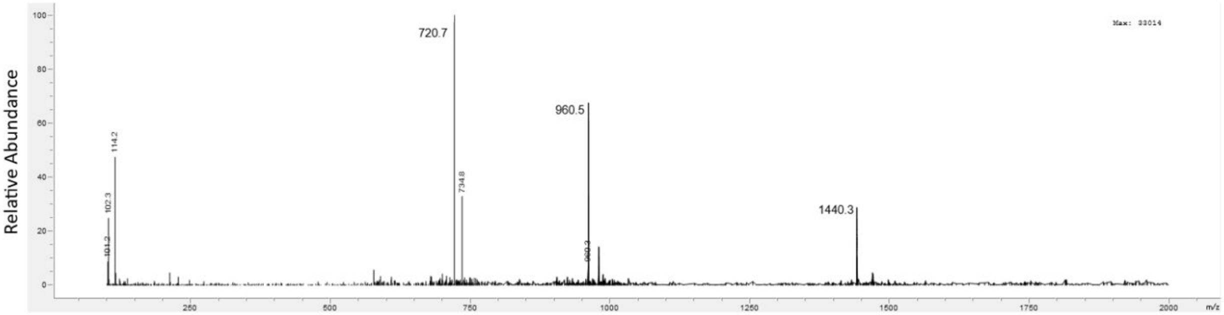

Supplemental Figure 6. Mass spectrum of SLG_2IGF.

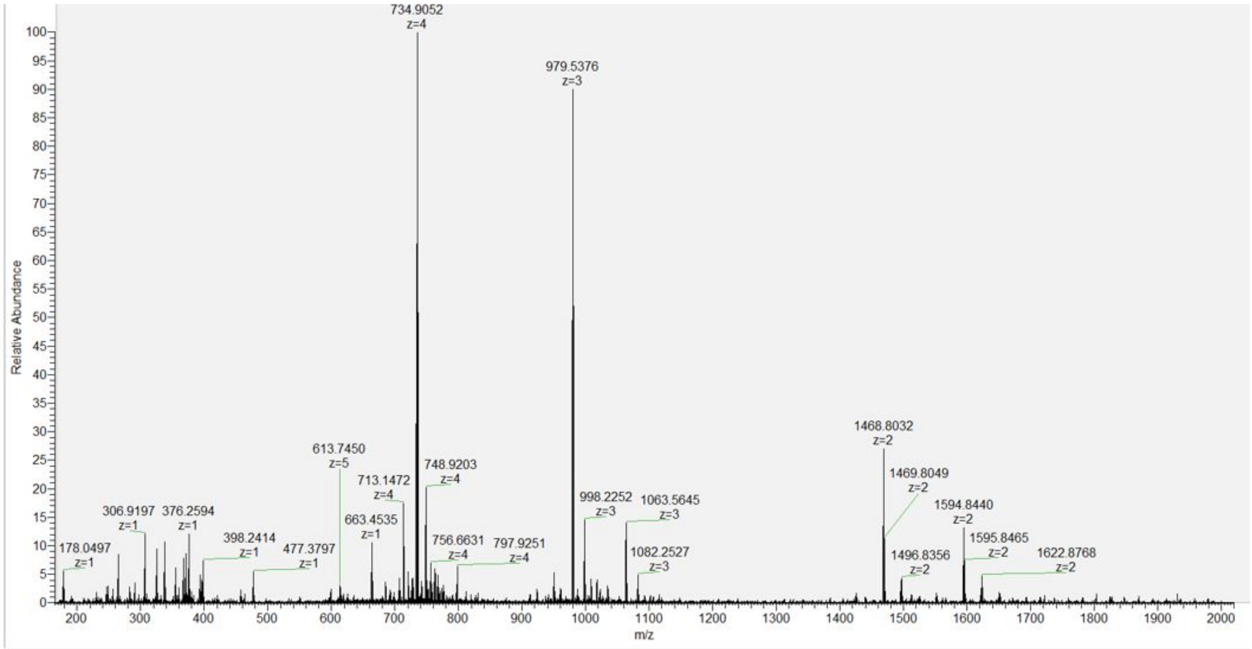

Supplemental Figure 7. Mass spectrum of SLG_3IGF.

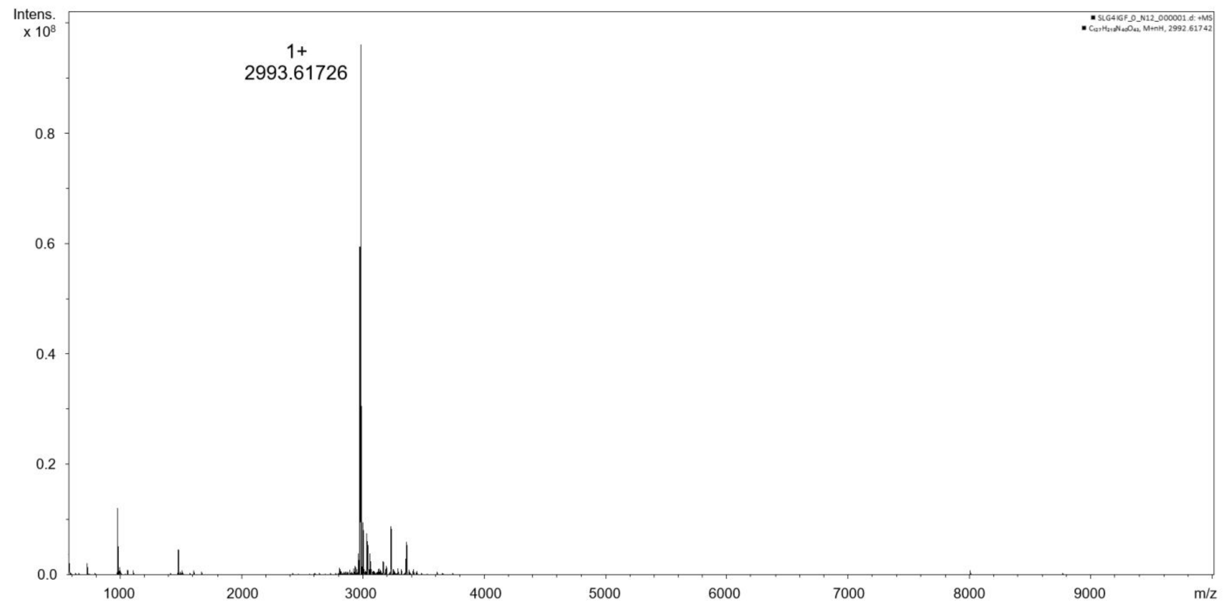

Supplemental Figure 8. Mass spectrum of SLG_4IGF.

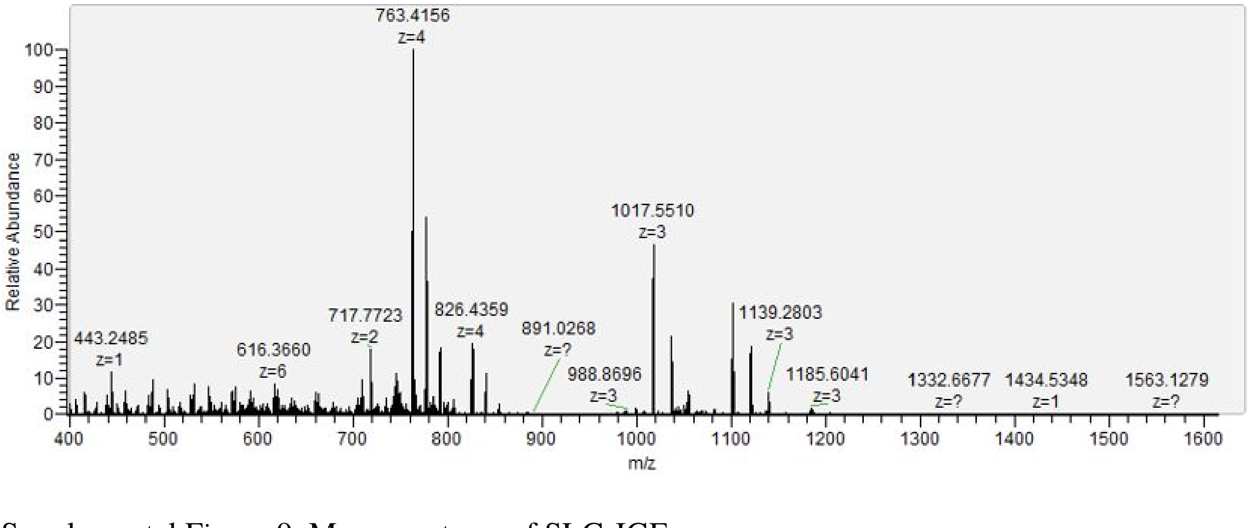

Supplemental Figure 9. Mass spectrum of SLG_5IGF.

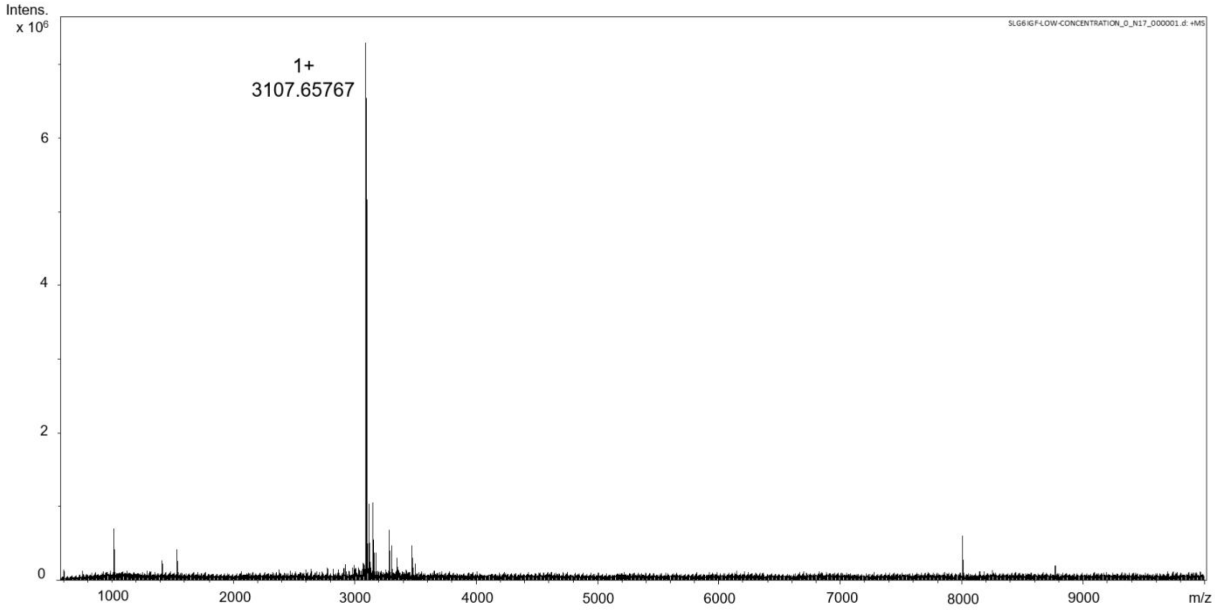

Supplemental Figure 10. Mass spectrum of SLG6IGF.

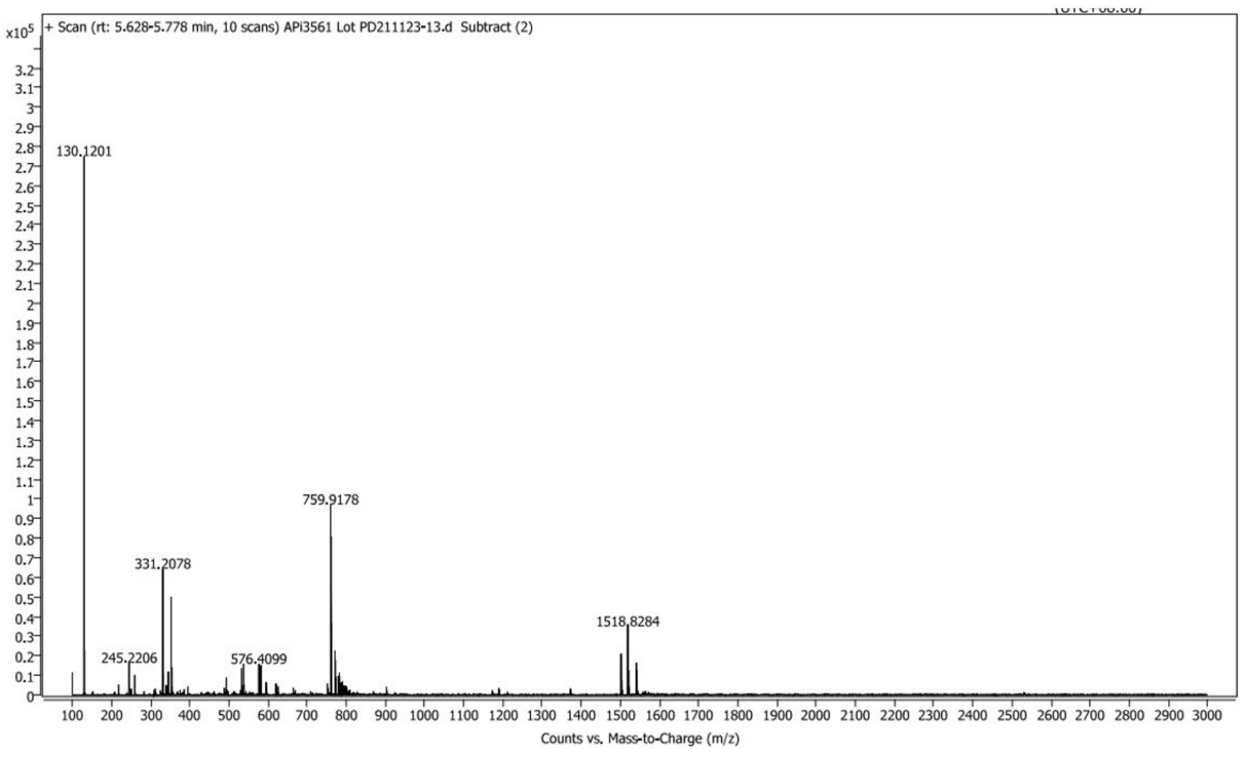

Supplemental Figure 11. Mass spectrum of E1 provided by AmbioPharm, Inc.

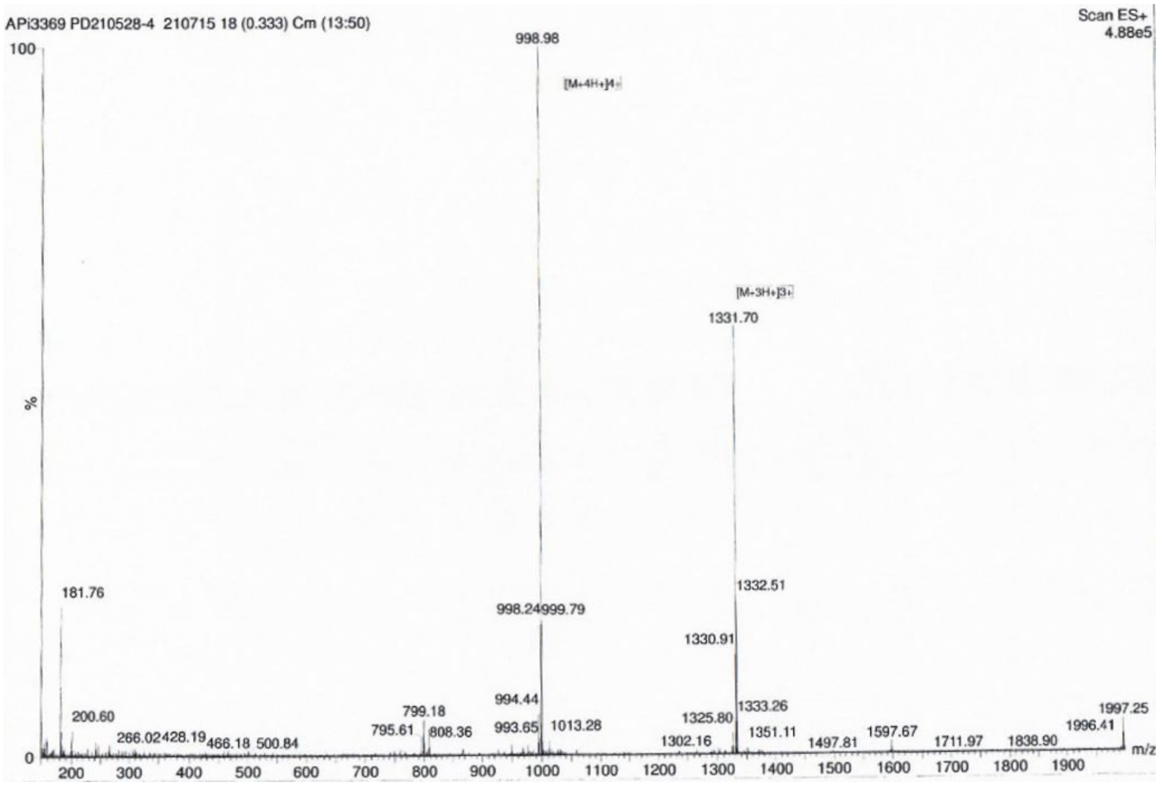

Supplemental Figure 12. Mass spectrum of ESBP3 provided by AmbioPharm, Inc.
